# Efficient method for *in situ* agitation of liquids directly inside NMR spectrometer

**DOI:** 10.1016/j.mex.2023.102254

**Published:** 2023-06-19

**Authors:** Antonio De Souza Braga Neto, Baptiste Rigaud, Guillaume Mériguet, Anne-Laure Rollet, Juliette Sirieix-Plénet

**Affiliations:** a Physicochimie des Electrolytes et Nanosystèmes interfaciaux (PHENIX), Sorbonne Université, 4 Place Jussieu, Paris F-75005, France; b Fédération de Chimie et Matériaux de Paris Centre, Sorbonne Université, 4 Place Jussieu, Paris F-75005, France

**Keywords:** Agitation method for samples inside NMR spectrometer, NMR, *In situ* agitation, Sample homogenisation, Localized spectroscopy (LOCSY), Liquid-liquid extraction, Ionic liquids

## Abstract

The objective of the method is to allow agitation and fast homogenization of liquid systems in NMR tubes, directly inside the NMR spectrometer. The setup makes it possible to record spectra of samples that are macroscopically not stable, as dispersions of large particles. It makes also possible to fasten the homogeneization of liquid during a reaction or a phase transition. In the present paper, the method has been evaluated using homogeneous liquid extraction (HLLE). This configuration can also be used to introduce gases in different systems to perform various types of experiments. The set up consists in a Teflon tube inserted in the NMR tube bringing gas that yields agitation by bubbling. The gas flow is tuned using an electronically operated valve connected to gas line and to the NMR console. The method details how to reach proper homogenization without any perturbation, as liquid leaks.

• An easy method for agitation of liquids inside NMR spectrometers.
• The set up can be used for the insertion of gases in the NMR tube inside the spectrometer.
• The method allows the study of the mixing of biphasic systems by NMR techniques.

## Graphical abstract

**Figure:**
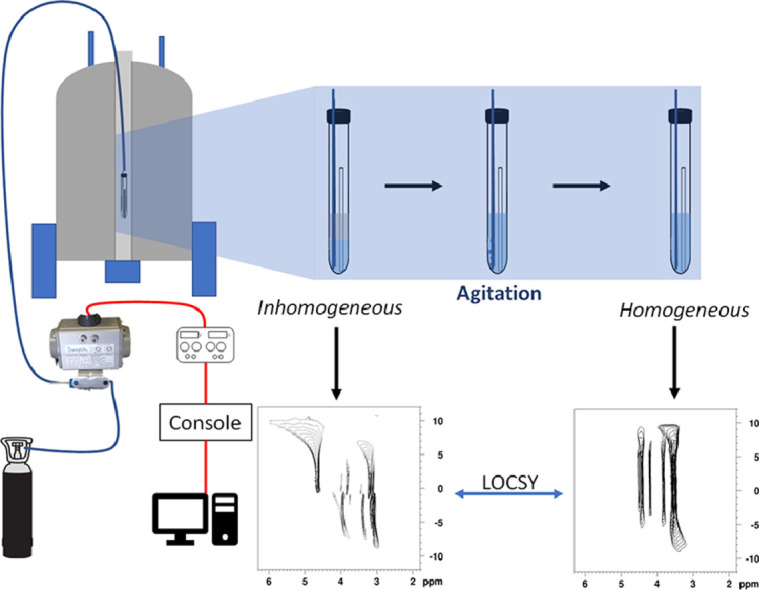

Specifications Table

**Table:** 

Subject area:	Chemistry
More specific subject area:	Nuclear Magnetic Resonance
Name of your method:	Agitation method for samples inside NMR spectrometer
Name and reference of original method:	N.A.
Resource availability:	N.A.

## Context

Nuclear magnetic resonance (NMR) spectroscopy is a useful tool used in different areas, from biology to materials. The homogeneity of the sample within the measurement zone is important to provide a clear insight into of the physico-chemical properties under investigation. However, the stability of samples at the macroscopic scale may not achieved. An illustrative example is the clay particle sedimentation in aqueous dispersion [1]. UltraFast NMR sequences [2,3] that allow the study of reacting systems, can partly circumvent the experimental hindrances of studying systems under evolution. Nevertheless, they cannot be applied in all cases, as for instance when signal to noise ratio is low. Then, agitation of the sample has to be performed to allow longer acquisition. Conversely, in systems where chemical reactions or phase transition occur, it may be crucial to promote the agitation of sample. Some authors have described, for instance, systems for evaluating microbial culture in which culture medium was circulated by rotary pumps inside the NMR spectrometer and for high-pressure analyses with benchtop NMR.

Although these methods can be used to promote agitation and gas insertion inside NMR spectroscopy, they require several accessories [4,5].

An important example in the domain of wastes recycling is the homogeneous liquid extraction (HLLE). Critical metals, such as rare earths, are extracted from acid aqueous solutions to a thermomorphic ionic liquid thanks to biphasic-monophasic transitions [6,7]. Depending on the temperature, mixtures of thermomorphic ionic liquids and aqueous solutions can indeed, be either biphasic or monophasic. Obviously, the removal of the interface at the monophasic state tremendously fastens the transport of critical metals from one phase to the other. However, this biphasic-monophasic transition is limited by diffusion. For *in situ* NMR study of such systems, there are presently two options. The first one is to heat the mixture inside the spectrometer and wait for the equilibrium, which is excessively time consuming. The second option is to agitate, outside the spectrometer, the mixture during the heating or cooling times, and after to insert it in the spectrometer. In addition to be not convenient, the temperature is not controlled during the transfer of the tube. It appears evident that an easy-to-use setup for *in situ* agitation inside the spectrometer has to be developed.

For the study of liquid-liquid extraction, slice selective NMR experiments are particularly worthwhile. They make it possible to record spectra at different positions/voxels within the sample, in other words give access to *in situ* spectral mapping, that are highly valuable information for a better understanding of the process.

This work uses for its demonstration, a radio frequency selective pulse method, the NMR localized spectroscopy LOCSY sequence [8]. The validation has been carried out using slice-selective NMR experiments offer a rapid and easy technique to obtain quantitative data on spatial distribution of the different species and to monitor it over time [9]. Nonetheless, artefacts can be observed in biphasic systems due to change in the magnetic susceptibility near the interfaces (liquid/liquid, liquid/air, liquid/glass). The presence of a refocusing pulse surrounded by two spoil gradients can greatly reduce this kind of artifacts [8,^10^,11].

## Methods details

### Homogenization setup

**Fig. 1:**
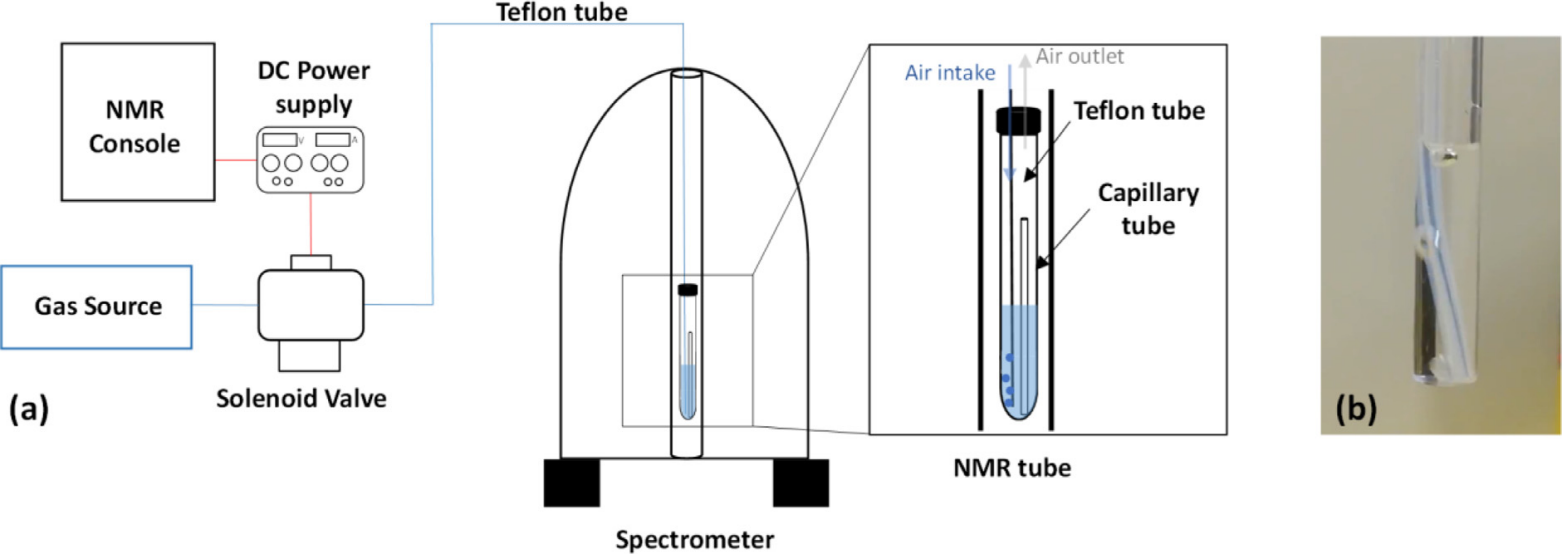
Agitation setup components and scheme. The complete schema of the agitation setup is presented in (a)The gas bubbling inside the NMR tube can be seen in (b).

The schema of the system and their components are presented in Fig. 1. The opening of the solenoid valve (Swagelok sc00015) allows the release of gas bubbles (N_2_) through a Teflon tube located inside the NMR tube. The solenoid valve is itself connected to a compressed gas line allowing a quickly switch. Its power supply is provided by a 24 V DC current (Iso-Tech IPS 2303DD). The spectrometer software (Topsin 3.6.2) controls the switching of the solenoid valve via a 1 V electrical signal which is retrieved from the Bruker Avance III NMR spectrometer console. This electrical signal acts as a switch to control the closing and opening of the solenoid valve. The electrical signal is driven by a specific pulse program. To activate the gas outlet, the console transmits a current to open the valve, which allows the injection of gas into the tube. The pulse program used is shown below.

1 ze

30 s setnmr3ˆ20

2 30 m

30 s setnmr3|20

exit

The pulse program instruction to turn the electrical output signal on or off is setnmr. The circumflex (gas in tube) and vertical bar (no gas flow) characters define the activation or deactivation of the 1 V signal, respectively. The setnmr instruction must be specified behind a delay. The bubble time is therefore defined between the two setnmr instructions. In particular, it is set to 30 min in this program. In this way, it is possible to correlate precisely the stirring time and the recording of the NMR spectra. A macro controls a series of different programs and pulse programs. In this macro, in a first step, the gas injection is deactivated, to avoid the effects of disturbances caused by the gas flow in the tube and consequently the inhomogeneity of the magnetic field. In a second step, it start the pulse program which determines the duration of the gas passage inside the tube during a given time. After this time of agitation, it turns off again to performs automatic tuning, shimming, and finally executes the NMR experiment. The gas flux is controlled manually in the gas line outlet and in the valve, in a way to provide the appropriate agitation for the studied systems, Fig. 1(b) presents an image of the gas flow inside the NMR tube. The complete schema of the agitation setup can be seen in Fig. 1(a).

### NMR tube preparation

A 10 mm tube was used in order to have enough space to fit both the Teflon tube and the glass capillary for the lock of the magnetic field. The different components are introduced in the following order: (1) the ionic liquid that is the densest phase, (2) the sealed capillary of 1.7 mm diameter, filled with deuterium oxide and (3) the aqueous phase. Two holes were made in the NMR tube cap, one larger of approximately 2.5 mm to insert the Teflon tube of outer diametre of 3.17 mm and an inner diametre of 1.58 mm, for the intake of gas, and finally one smaller of 0.5 mm to work as gas outlet. To avoid capillarity effects inside the Teflon tube, and in consequence the formation of interfaces at different positions in the NMR tube, the Teflon tube was perforated at several points in the part which is immersed in the ionic liquid.

**Fig. 2:**
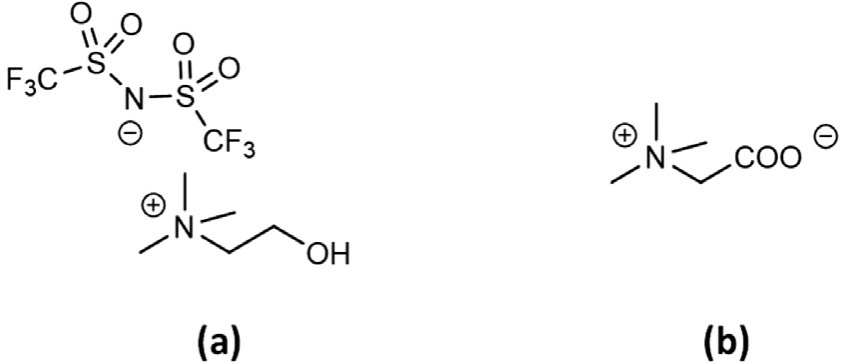
Chemical structure of (a) [Chol][TFSI] and (b) betaine.

The method proposed in this work is validated using a HLLE system. The thermomorphic ionic liquid used here is cholinium bis(trifluoromethyl-sulfonyl)imide, [Chol][TFSI]. The aqueous solution contains betaine (trimethyl-glycine). Fig. 2 presents the chemical structure of the different molecules. [Chol][TFSI] ionic liquid and water, in a mass fraction of 1:1, form a biphasic system at room temperature, and become monophasic above 72 °C, its upper critical transition temperature [12]. The betaine in this system plays the role of metal extractant. Note that for the method validation, no metal is necessary and therefore none has be introduced in the aqueous phase.

### NMR spacial resolution of a sample

The LOCSY (LOCalized SpectroscopY) has been proposed by Mantel et al. [8] to study different species in samples that are heterogeneous at macroscopic scale. The essence of the sequence is similar to STEAM (STimulated Echo Acquisition Mode) sequence [13], although the spacial resolution is only achieved along one direction. In the LOCSY sequence, a pulsed field gradient is applied on the vertical axis (*z*), i.e. along the NMR tube, allowing the spacial encoding of a sample in this direction. The spins therefore experience different magnetic fields as *B*(*z*) = *B*_0_ + *Gz*, where *G* is the strength of the applied gradient, *B*_0_ the static magnetic field and *z* the position. As a result the resonance frequencies, *ω*, varies along the direction *z* according to Eq. (1). Simultaneously a frequency selective excitation pulse is applied, which excites only selected a narrow frequency range, and thus only a spatial slice of the sample.

(1) $$\omega \;\left(z\right)=\frac{\gamma }{2\pi }\left[B_{0}+G_{z}\right]$$

where *γ* is the gyromagnetic ratio.

The slice size ∆*z* of the sample is defined by the excitation pulse bandwidth *BW* according Eq. (2) [[8], [14]] It is important to notice that the size of slice is limited by the gradient strength *G*.

(2) $$\Delta_{z}=\frac{\mathrm{BW}}{\gamma G}$$

The maximum area of the sample that can be observed corresponds to the size of the coil. Regions outside the coil detection zone cannot be observed.

**Fig. 3:**
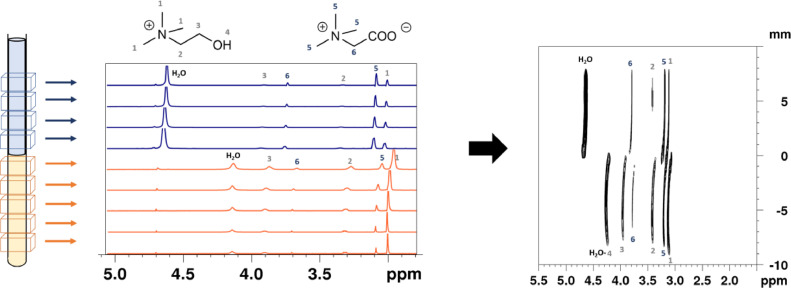
LOCSY slice-selective spectral study of thermomorphic system. Proton NMR 1D series of spectra obtained along the *z*-axis of the tube and the resulting 2D NMR map. The NMR resonances assignments are indicate by numbers. The peak around 4.7 ppm corresponds to D_2_O in the capillary tube.

Fig. 3 shows the proton NMR 1D series of spectra obtained along the *z*-axis of the tube and the resulting 2D NMR map. The origin (*z* = 0) was set at the position of the liquid-liquid interface at room temperature. The compositions of the two phases are well observed, along with the interface of the system. The bottom half of the 2D map contains the signal of the ionic liquid phase. Note that [Chol][TFSI] is saturated with water and also contains some betaine, that has diffused from the aqueous phase. Also the peaks are shifted to the right in the ionic liquid compared to the aqueous phase.

To obtain this map a pulsed field gradient of 5.5 G/cm was used, corresponding to 10% of gradient-amplifier power in a 5 mm BBI probe. A refocusing pulse of RE-BURP type with bandwidth of 1170 Hz was applied. The frequency offset of the pulses was incremented in steps of 1170 Hz from −19890 to +19890 Hz. It corresponds to slices of 1000 µm. The experiments were performed using a 500 MHz Bruker Avance III spectrometer equipped with a 5 mm BBI probe with a maximum 55 G/cm unixial *z* gradient or a 10 mm BBO probe with *z* gradient. All data were obtained and processed using Bruker TOPSPIN—NMR software.

## Method validation

**Fig. 4:**
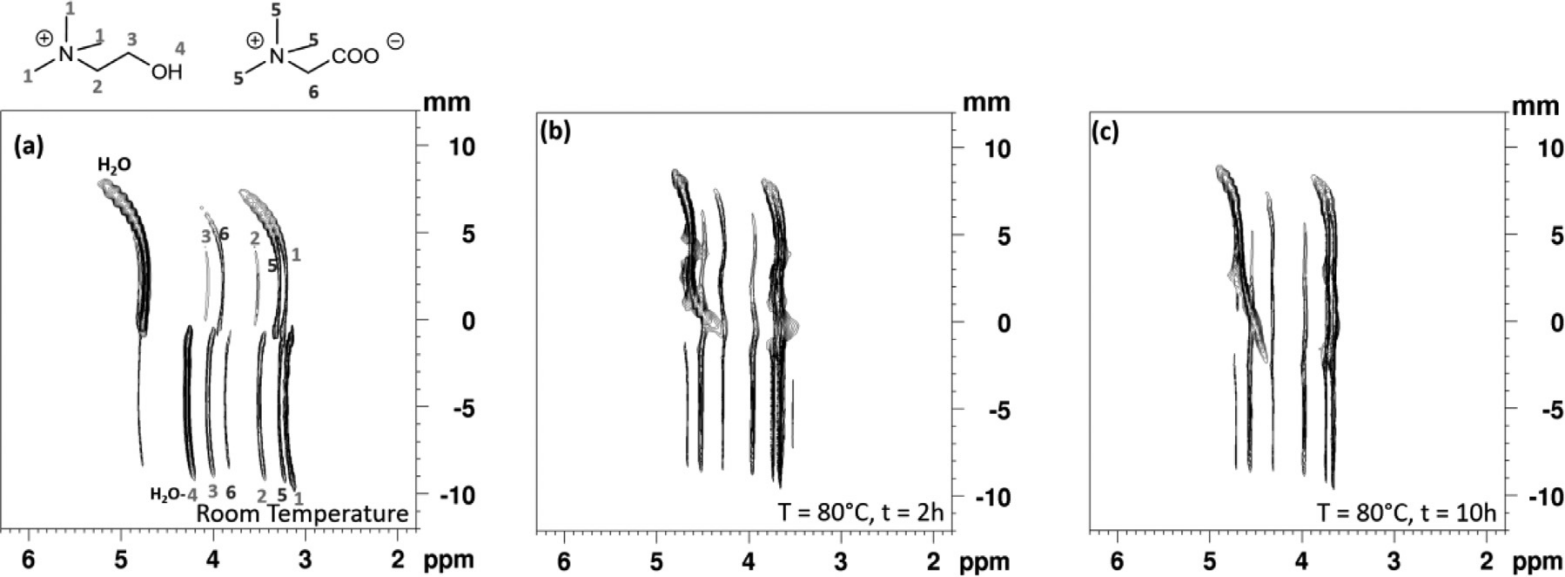
LOCSY 2D map of [Chol][TFSI] and aqueous phase biphasic system at (a) room temperature (b) heated at 80 °C after 2 h and, (c) after 10 h at 80 °C. The maps were obtained with a pulsed field gradient of 5.5 G/cm was used, corresponding to 10% of gradient-amplifier power in a 5 mm BBI probe. A refocusing pulse of RE-BURP type with bandwidth of 1170 Hz was applied. The frequency offset of the pulses was incremented from −30420 to +35100 Hz in steps of 1170 Hz. Some resonance assignments are indicated by the numbers.

To validate the method, we have performed a LOCSY experiment on the HLLE system with and without agitation. To follow the biphasic to monophasic transition of the thermomorphic system, [Chol][TFSI] and an aqueous phase, it is necessary to increase the temperature of the system from room temperature up to 80 °C. First, Fig. 4(a) shows the resulting NMR 2D map of the biphasic system at room temperature, the top of the map part corresponds to the aqueous phase of the system and the bottom part to the ionic liquid phase, as described before. Nevertheless, these spectra were recorded with a wider range of offset frequencies than in Fig. 3. The resulting empty upper and lower areas of the 2D maps is outside the coil thus explaining the absence of signal. A shift of resonances and a broadening are also observed at the interface of biphasic system, at the meniscus and at the extremities of the coil. Magnetic susceptibility variations might be the cause of these observations. The magnetic field heterogeneity can be difficult to be fully compensated by magnet shimming. Second, Fig. 4(b) and (c) show the system at 80 °C without agitation for 2 h and 10 h, respectively. A shift of resonances and the start of phases mixing are observed. Despite these long heating times complete mixing of phases is not seen and the sample remains heterogeneous even after more than 20 h under 80 °C (data not shown). This fact confirms the absence of convection on the systems, since otherwise it would yield a faster biphasic to monophasic transition.

**Fig. 5:**
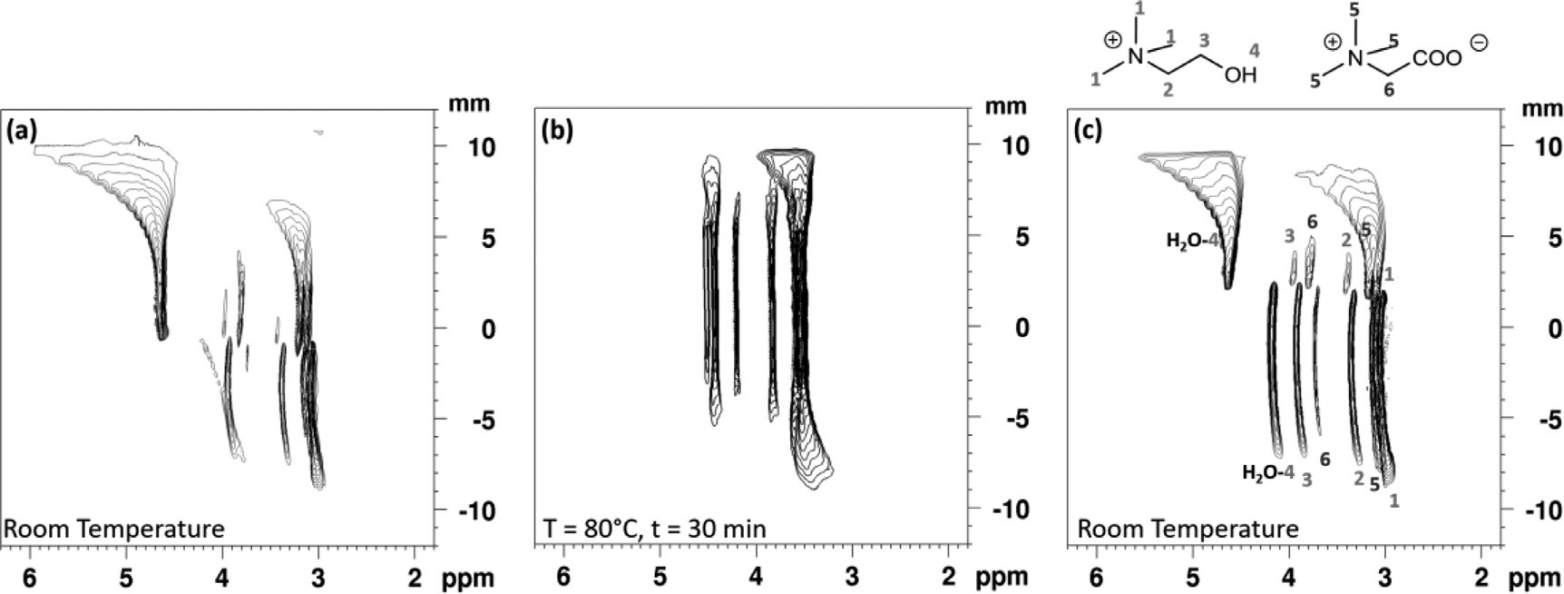
LOCSY 2D map of [Chol][TFSI] and aqueous phase biphasic system at (a) room temperature, (b) heated at 80 °C after agitation during 30 min, (c) return at room temperature after approximately 4 h. The vertical axis origin was arbitrarily set to the position of the interface. The maps were obtained with a pulsed field gradient of 5.5 G/cm was used in a 10 mm BBO probe. A refocusing pulse of RE-BURP type with bandwidth of 1170 Hz was applied. The frequency offset of the pulses was incremented from −10800 to +6750 Hz in steps of 450 Hz, equivalent to 400 µm slices.

Fig. 5 presents the same system but with the homogenization setup, which permits the agitation of the sample inside the spectrometer. The acquisition, at room temperature and without agitation, is started just right after the contact with the two phases, IL and aqueous phase (Fig. 5(a)). For this reason, the betaine diffuses progressively during the acquisition from the aqueous phase into the ionic liquid phase. In consequence, a shift of resonances of the OH—H_2_O peak (see the fork shape around 4 ppm in Fig. 5(a)) in the IL phase along the *z*-axis in the NMR tube, is observed. This change is related with the concentration difference of the betaine in the IL phase. This shift means that the betaine concentration is higher close to the interface.

**Fig. 6:**
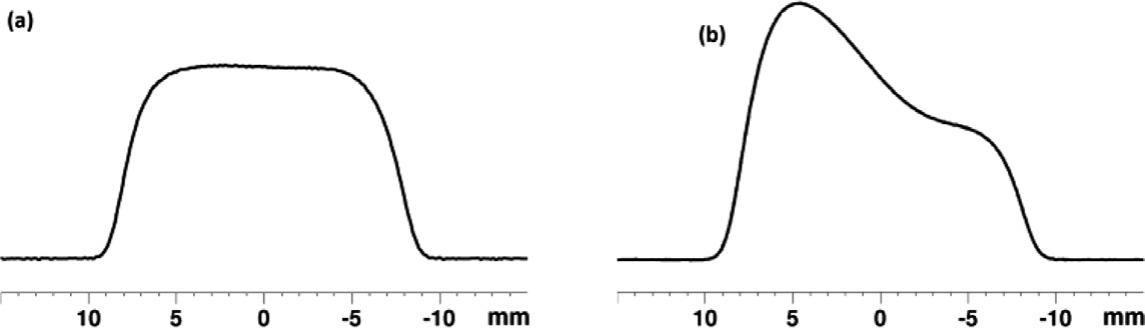
Echograd spectra of the sample heated at 80 °C after (a) 30 min of agitation and (b) 20 h, without agitation setup.

In a second step, the system is heated at 80 °C and the system reaches this temperature within 15 min. The agitation starts immediately when the temperature reaches 80 °C. Notably, after only 30 min of agitation, the complete mixing of the phase and the homogenization are achieved (Fig. 5(b)). The homogenization of the system can be also confirmed with a 1D image of the sample acquired by an echograd sequence [15]. This experiment makes it possible to obtain 1D image of the sample. Fig. 6(a) shows the Echograd profile of the tube at 80 °C after agitation. The plateau observed confirms a homogeneous distribution all over the sample. On the contrary, without agitation a heterogeneous distribution is observed even after 20 h at 80 °C (Fig. 6(b)). Furthermore, the echograd indicates that no convection occurs at high temperature. Note that the edges of the spectra corresponding to the edge effects of the coil.

The return of the sample to room temperature is observed in Fig. 5(c). Due to the mass transfer between the phases during the process, a change in the interface height occurs. Resonance shifts and peak broadening are also observed in the spectra on Fig. 5 due to the boundaries of the excitation volume of the coil.

### Recommendations

To insert the NMR tube in the spectrometer, the air lift cannot be used alone but the Teflon tube has to be guided carefully through the bore in a way that allows the NMR tube to go down to the probe, avoiding that the tube stays stuck in the upper parts of the spectrometer bore. The hole in the NMR tube stopper must be tight enough to keep the Teflon tube fixed in the NMR tube. Once the experiment is done, and it might be necessary to pull gently the Teflon tube in order to lift the NMR tube. For these reasons a flexible tube that doesn't interfere with the NMR signal is recommended in this method.

The macro was used to facilitate the experiments, but it is not necessary, only the pulse program to operate the valve is required, although the use of a macro to unlock during agitation, lock, shim and tune after the agitation is convenient for the user.

For the method validation presented in this work, no precise measure of the gas flow rate was done, and it can variate in the function of the properties of the system analysed and objectives. However, making a small vent in the stopper for the gas outlet and checking the gas flow inside the NMR tube visually before inserting it in the spectrometer is fundamental to prevent liquid projections inside the spectrometer.

## Conclusion

This method makes it possible to successfully perform agitation thanks to gas bubbling setup in the NMR tube itself located inside the spectrometer and hence to homogenize the sample. The validation of our method has been carried using homogeneous liquid-liquid extraction processes and slice-selective NMR experiments. It has been evidenced that clean homogenization was rapidly achieved, whereas without agitation, the system is still heterogeneous after 20 h. This method can be applied for various purposes such as macroscopically non stable samples, or to insert operando gas within liquid sample.

## CRediT authorship contribution statement

**Antonio De Souza Braga Neto:** Investigation, Methodology, Conceptualization, Writing – original draft, Writing – review & editing. **Baptiste Rigaud:** Investigation, Methodology, Conceptualization, Writing – original draft, Writing – review & editing. **Guillaume Mériguet:** Investigation, Methodology, Conceptualization, Writing – original draft, Writing – review & editing. **Anne-Laure Rollet:** Investigation, Methodology, Conceptualization, Writing – original draft, Writing – review & editing. **Juliette Sirieix-Plénet:** Investigation, Methodology, Conceptualization, Writing – original draft, Writing – review & editing.

## Declaration of Competing Interest

The authors declare that they have no known competing financial interests or personal relationships that could have appeared to influence the work reported in this paper.

## Data Availability

Data will be made available on request.

## References

[1] Fanost A., Jaber M., de Viguerie L., Korb J.P., Levitz P.E., Michot L.J., Mériguet G., Rollet A.L. (2021). Green earth pigments dispersions: water dynamics at the interfaces. J. Colloid Interface Sci..

[2] Mankinen O., Zhivonitko V.V., Selent A., Mailhiot S., Komulainen S., Prisle N.L., Ahola S., Telkki V.V. (2020). Ultrafast diffusion exchange nuclear magnetic resonance. Nat. Commun..

[3] Giraudeau P., Frydman L. (2014). Ultrafast 2D NMR: an Emerging Tool in Analytical Spectroscopy. Annu. Rev. Anal. Chem..

[4] Duchowny A., Dupuy P.M., Christin Widerøe H., Berg O.J., Faanes A., Paulsen A., Thern H., Mohnke O., Ku¨ppers M., Blu¨mich B., Adams A. (2021). Versatile high-pressure gas apparatus for benchtop NMR: design and selected applications. J. Magn. Reson..

[5] Noguchi Y., Shimba N., Toyosaki H., Ebisawa K., Kawahara Y., Suzuki E., Sugimoto S. (2002). In vivo NMR system for evaluating oxygen-dependent metabolic status in microbial culture. J. Microbiol. Methods.

[6] Vander Hoogerstraete T., Onghena B., Binnemans K. (2013). Homogeneous liquid-liquid extraction of metal ions with a functionalized ionic liquid. J. Phys. Chem. Lett..

[7] Onghena B., Jacobs J., Meervelt L.V., Binnemans K. (2014). Homogeneous liquid-liquid extraction of neodymium(III) by choline hexafluoroacetylacetonate in the ionic liquid choline bis(trifluoromethylsulfonyl)imide. Dalton Trans..

[8] Mantel C., Bayle P.A., Hediger S., Berthon C., Bardet M. (2010). Study of liquid- liquid interfaces by an easily implemented localized NMR sequence. Magn. Reson. Chem..

[9] Pantoja C.F., Muñoz-Muñoz Y.M., Guastar L., Vrabec J., Wist J. (2018). Compo- sition dependent transport diffusion in non-ideal mixtures from spatially resolved nuclear magnetic resonance spectroscopy. Phys. Chem. Chem. Phys..

[10] Kozminski W. (2000). Application of spatially resolved NMR spectrostopy for high resolution spectra of het- erogeneous samples. Pol. J. Chem..

[11] Mitrev Y.N. (2017). Slice selective NMR approach for investigation of distribution phenomena in biphasic samples. Bulg. Chem. Commun..

[12] Nockemann P., Binnemans K., Thijs B., Parac-Vogt T.N., Merz K., Mudring A.V., Menon P.C., Rajesh R.N., Cordoyiannis G., Thoen J., Leys J., Glorieux C. (2009). Temperature-driven mixing-demixing behavior of binary mixtures of the ionic liquid choline bis(trifluoromethylsulfonyl)imide and water. J. Phys. Chem. B.

[13] Lambert J., Hergenröder R., Suter D., Deckert V. (2009). Probing liquid-liquid interfaces with spatially resolved NMR spectroscopy. Angew. Chem. Int. Ed..

[14] Dumez J.N. (2018). Spatial encoding and spatial selection methods in high-resolution NMR spectroscopy. Prog. Nucl. Magn. Reson. Spectrosc..

[15] Júnior L.H.K.Queiroz, Ferreira A.G., Giraudeau P. (2013). Optimization and practical imple- mentation of ultrafast 2D NMR experiments. Quím. Nova.

